# Expression of *NOTCH1* Is Correlated with Expression of Cancer Stem Cell Markers and *miR-150* in Oral Epithelial Dysplasia

**DOI:** 10.3390/ijms27041946

**Published:** 2026-02-18

**Authors:** Emanuela Boštjančič, Gašper Grubelnik, Nina Zidar, Katarina Dimnik

**Affiliations:** Institute of Pathology, Faculty of Medicine, University of Ljubljana, 1000 Ljubljana, Slovenia; emanuela.bostjancic@mf.uni-lj.si (E.B.); gasper.grubelnik@mf.uni-lj.si (G.G.); nina.zidar@mf.uni-lj.si (N.Z.)

**Keywords:** *NOTCH1*, microRNAs, stem cell markers, squamous cell carcinoma, oral epithelial dysplasia

## Abstract

NOTCH1 is associated with various tumors, including oral squamous cell carcinoma (OSCC), with a complex role depending on cellular contexts. Our aim was to analyze the expression of NOTCH1, several stem cell markers, and selected microRNAs in preneoplastic lesion of the oral cavity, oral epithelial dysplasia (OAD). Our study included formalin-fixed paraffin-embedded biopsy samples of 36 cases of OAD and 15 cases of normal oral mucosa. Expression of *NOTCH1*, stem cell markers (*AGR2*, *KLF4*, *NANOG*, *OCT4*, *SOX2*), and *miR-27a*, *miR-34a*, *miR-128*, *miR-145*, *miR-150*, and *miR-335* was analyzed by quantitative PCR (qPCR). Expression of NOTCH1 protein was analyzed by immunohistochemistry. In OAD compared to normal mucosa, we found a significant increase in mRNA levels of *NOTCH1*, stem cell markers *AGR2*, *NANOG*, *OCT4*, and *SOX2*, and *miR-150* and *miR-128*. *NOTCH1* mRNA positively correlated with all five stem cell markers’ mRNA levels and *miR-150*. Immunohistochemistry showed variable expression patterns of NOTCH1 in OAD and normal mucosa. Our results support the role of NOTCH1 in early phases of OSCC development, with a potential contributory role in stemness, in association with *AGR2*, *NANOG*, *OCT4*, and *SOX2*, *miR-150* and *miR-128*. These results support a complex role of NOTCH1 in carcinoma development, i.e., from oncogenic to tumor suppressor roles and stemness maintenance, not only in invasive OSCC but also in its precursor—OED.

## 1. Introduction

NOTCH1 is a transmembrane receptor protein involved in the Notch signaling pathway, determining cell fate, survival, and proliferation during physiological processes and in various diseases, including tumors [1]. Changes in Notch signaling have been associated with the initiation and progression of various malignancies. The Notch pathway plays a complex role depending on the cellular context, and it has been demonstrated to be involved in various aspects of tumor biology such as angiogenesis, metabolic programming, tumor microenvironment, epithelial–mesenchymal transition (EMT), abnormal activation patterns, and expression of stem cell markers [2].

*NOTCH1* mutations in squamous cell carcinoma (SCC) of the head and neck are believed to result mostly in loss of function of NOTCH1, giving NOTCH1 a tumor suppressor role in this type of cancer [3]. On the other hand, up-regulation of NOTCH1, proposing an oncogenic role, has also been described in oral SCC (OSCC) [4,5,6]. Better understanding of NOTCH1 signaling in OSCCs is crucial for clinically targeting this pathway. The presence of *NOTCH1* mutations may predict response to treatment with immune checkpoint inhibitors or phosphatidylinositol 3-kinase inhibitors [7]. It is believed that NOTCH1 also contributes to stemness and is involved in EMT, which is considered to be one of the processes involved in the formation of cancer stem cells (CSCs) [8]. CSCs are a small fraction of tumor cells involved in tumor development and propagation due to their ability for self-renewal, differentiation into different cell types, and mobility. They are mostly found as a subpopulation at the invasive tumor front. CSCs are well described as contributors to development and invasiveness of OSCC [9].

The pathogenesis of invasive OSCC is a complex process involving various molecular and morphologic events. OSCC often develops from preexisting dysplasia. Oral epithelial dysplasia is defined as a spectrum of epithelial architectural and cytological changes that result from the accumulation of molecular alterations, with an increased risk of transformation into invasive SCC [10,11,12]. Despite advances in understanding the development and progression of OSCC, 5-year survival rates remain in the range of 50–60% [13]. Thorough investigation of molecular background of OSCC—especially in its precursor lesion (oral epithelial dysplasia)—is therefore still of high interest for the identification of potential markers not only for treatment, but also for prevention and early detection [11,12].

Results of our previous studies are in accordance with the complexity of NOTCH1 in invasive OSCC and with the potential role of NOTCH1 in cancer stemness in OSCC. Namely, we observed that expression of *NOTCH1* was correlated with CSC markers, for example, *KLF4*, *NANOG*, and *OCT4*, and with the expression of microRNAs (miRNAs) *miR-27a*, *miR-34a*, *miR-150*, *miR-145*, and *miR-335* in OSCC [14]. These results suggest that NOTCH1 signaling is dependent on many regulatory factors, including CSCs and miRNAs. Interestingly, there are limited data in the literature indicating that these miRNAs might regulate or be regulated by *NOTCH1*, but the majority of them have been reported to play a role in various cancers, including OSCC [15].

Although NOTCH1 is relatively well investigated in OSCC, its expression, correlation with its potential regulators and its regulatory function in CSCs are poorly understood in early stages of OSCC development, i.e., dysplasia. We therefore analyzed the expression of NOTCH1 in correlation with the expression of stem cell markers, (*AGR2*, *KLF4*, *NANOG*, *OCT4*, *SOX2*) and previously identified miRNAs (*miR-27a*, *miR-34a*, *miR-128*, *miR-150*, *miR-145*, *miR-335*) in oral epithelial dysplasia.

## 2. Results

### 2.1. Expression of NOTCH1 mRNA in Oral Epithelial Dysplasia and Normal Oral Mucosa

**Figure 1:**
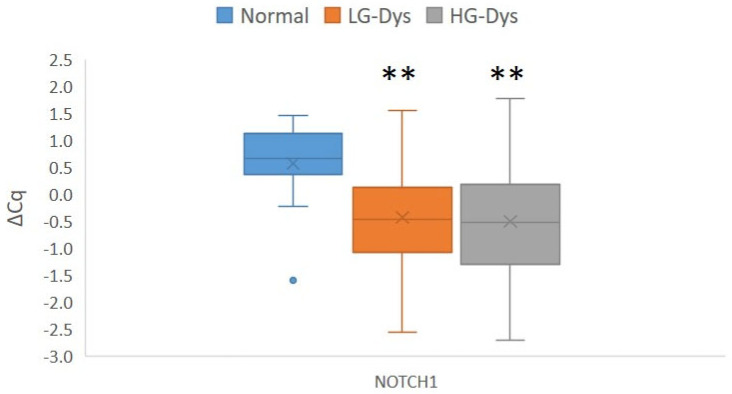
Expression of *NOTCH1* in normal oral mucosa and oral epithelial dysplasia. Legend: ΔCq, delta Cq; Cq, quantitation cycle; Dys, dysplasia; LG, low-grade; HG, high-grade; **, *p* < 0.01 (Mann–Whitney U test), statistically significant after correction for multiple comparisons. Number of samples: HG-Dys, *n* = 36; LG-Dys, *n* = 36; Normal, *n* = 15.

We found up-regulation of *NOTCH1* mRNA in low-grade dysplasia (LG dysplasia) and high-grade dysplasia (HG dysplasia) (*p* = 0.001 for both) in comparison with normal oral mucosa. There were no differences in the expression of *NOTCH1* mRNA between LG and HG dysplasia. Results are summarized in Figure 1.

### 2.2. Expression of Stem Cell Markers AGR2, KLF4, NANOG, OCT4, SOX2 in Oral Epithelial Dysplasia and Normal Oral Mucosa

**Figure 2:**
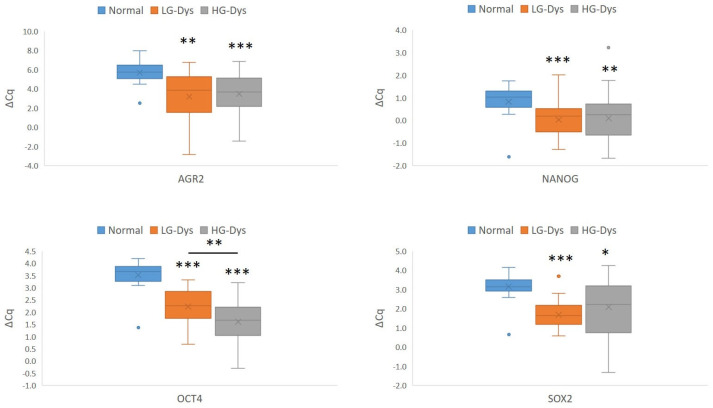
Expression of stem cell markers (*AGR2*, *NANOG*, *OCT4*, *SOX2*) in normal oral mucosa and oral epithelial dysplasia. Legend: ΔCq, delta Cq; Cq, quantitation cycle; Dys, dysplasia; LG, low-grade; HG, high-grade; *, *p* < 0.05; **, *p* < 0.01; ***, *p* < 0.001 (Mann–Whitney U test); ** and ***, statistically significant after correction for multiple comparisons. Number of samples: HG-Dys, *n* = 36; LG-Dys, *n* = 36; Normal, *n* = 15.

In LG dysplasia, in comparison with normal mucosa, up-regulation of all CSC markers except *KLF4* was found: *AGR2* (*p* = 0.001), *NANOG* (*p* < 0.001), *OCT4* (*p* < 0.001), and *SOX2* (*p* < 0.001). In HG dysplasia, in comparison with normal mucosa, up-regulation of all CSC markers except *KLF4* was observed: *AGR2* (*p* < 0.001), *NANOG* (*p* = 0.002), *OCT4* (*p* < 0.001), *SOX2* (*p* = 0.011). Comparison of CSC marker expression between LG and HG dysplasia revealed up-regulation of *OCT4* in HG dysplasia compared with LG dysplasia, showing a gradual increase in expression from normal mucosa to HG dysplasia. Results of statistically differentially expressed CSC markers are summarized in Figure 2.

### 2.3. Expression of miRNAs miR-27a, miR-34a, miR-128, miR-145, miR-150, miR-335 in Oral Epithelial Dysplasia and Normal Oral Mucosa

**Figure 3:**
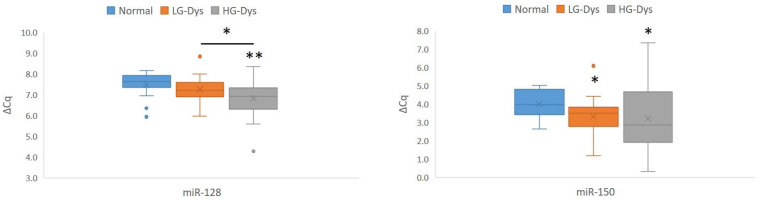
Expression of *miR-128* and *miR-150* in normal oral mucosa and oral epithelial dysplasia. Legend: ΔCq, delta Cq; Cq, quantitation cycle; Dys, dysplasia; LG, low-grade; HG, high-grade; *, *p* < 0.05; **, *p* < 0.01 (Mann–Whitney U test); **, statistically significant after correction for multiple comparisons. Number of samples: HG-Dys, *n* = 36; LG-Dys, *n* = 36; Normal, *n* = 15.

Comparison of miRNA expression between LG dysplasia and normal oral mucosa revealed up-regulation of *miR-150* (*p* = 0.021). Comparison of miRNA expression between HG dysplasia and normal mucosa revealed up-regulation of *miR-128* and *miR-150* (*p* = 0.003 and *p* = 0.028, respectively). Results of significantly differentially expressed miRNAs are summarized in Figure 3. There were no differences in *miR-27a* expression between the three groups. Whereas *miR-150* did not distinguish between LG and HG dysplasia, *miR-128* showed up-regulation in HG dysplasia when compared with LG dysplasia. Results of our analyses of expression of *miR-34a* and *miR-145* in oral epithelial dysplasia in comparison with normal mucosa were presented elsewhere [16].

### 2.4. Correlation Between Expression of NOTCH1, AGR2, KLF4, NANOG, OCT4, SOX2 mRNAs and miRNAs miR-27a, miR-34a, miR-128, miR-145, miR-150, miR-335 in Oral Epithelial Dysplasia and Normal Oral Mucosa

**Figure 4:**
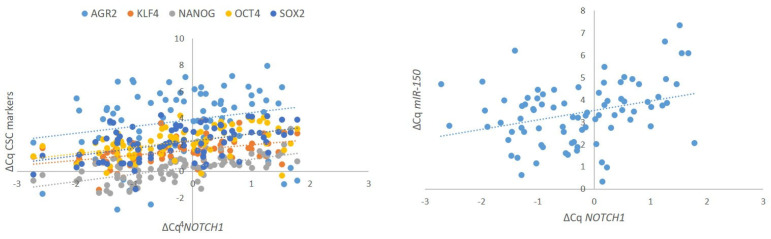
Correlation of expression of *NOTCH1* with CSC markers (*AGR2*, *KLF4*, *NANOG*, *OCT4*, *SOX2*) and *NOTCH1* with *miR-150* in oral epithelial dysplasia. Legend: ΔCq, delta Cq; Cq, quantitation cycle (Spearman’s Rank correlation coefficient test). Number of samples included: *n* = 87 (HG-Dys, *n* = 36; LG-Dys, *n* = 36; Normal, *n* = 10).

We found a strong positive correlation between *NOTCH1* and all five analyzed CSC markers, namely *AGR2* (r_s_ = 0.249, *p* = 0.025), *KLF4* (r_s_ = 0.479, *p* < 0.001), *NANOG* (r_s_ = 0.646, *p* < 0.001), *OCT4* (r_s_ = 0.554, *p* < 0.001), and *SOX2* (r_s_ = 0.436, *p* < 0.001). We also observed a strong positive correlation between the expression of *NOTCH1* and *miR-150* (r_s_ = 0.318, *p* = 0.004). Results are summarized in Figure 4. All correlations are included in Supplementary Table S1, with corresponding statistical power and estimation of required sample size in Supplementary Table S2.

### 2.5. Immunohistochemical Expression of NOTCH1 Protein in Oral Epithelial Dysplasia

**Figure 5:**
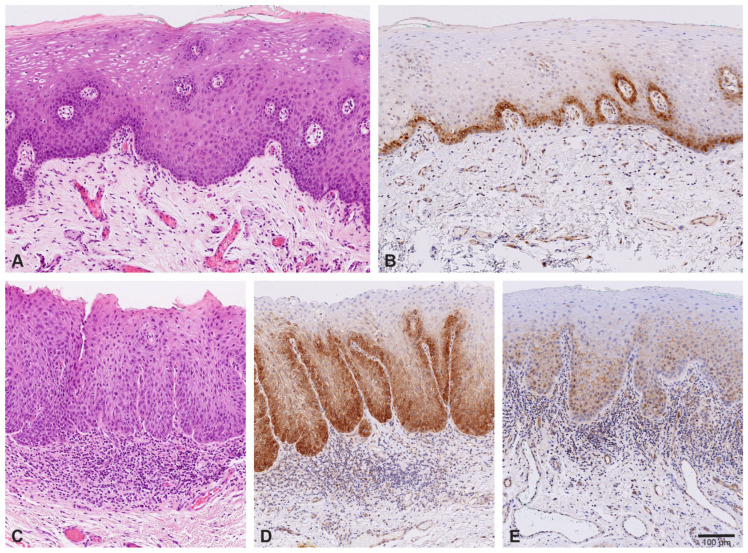
Immunohistochemical staining for NOTCH1 (magnification 10×). Normal oral mucosa (**A**) with positive staining for NOTCH1 in basal/parabasal cells (**B**). Oral epithelial dysplasia (**C**) with strong (**D**) and weak (**E**) staining for NOTCH1. Number of samples included: *n* = 56 (HG-Dys, *n* = 28; LG-Dys, *n* = 28).

Immunohistochemical analysis of NOTCH1 protein expression was performed on 28 cases of oral epithelial dysplasia and normal oral mucosa (Figure 5).

Reaction against NOTCH1 was cytoplasmic, membranous, and sometimes nuclear, often in combination. In addition to epithelial cells, staining of endothelial cells and some inflammatory cells was also observed (Figure 5B).

In oral epithelium with no signs of dysplasia, NOTCH1 was present in the basal/parabasal cells, often with quite intense staining (Figure 5B). However, the reaction was sometimes patchy, with areas of weak or negative staining, of uncertain significance.

In nine (39%) cases of oral dysplasia, the NOTCH1 expression score was higher than in normal mucosa, mostly due to an increased extent of the reaction in higher epithelial layers (Figure 5D). In 11 cases of dysplasia (48%), NOTCH1 expression score was similar (eight cases, 35%) or lower (three cases, 13%) than in normal mucosa; even though the extent of reaction was often increased, the intensity was lower (Figure 5E). In three (13%) cases of dysplasia, staining was completely negative or present only in rare cells, with staining of endothelial cells serving as a positive control.

Finally, we did not observe any association between mRNA expression of NOTCH1, other stem cell markers, or miRNAs and immunohistochemical expression of the NOTCH1 protein.

## 3. Discussion

We analyzed the expression of NOTCH1, stem cell marker mRNAs *AGR2*, *KLF4*, *NANOG*, *OCT4* and *SOX2*, and miRNAs *miR-27a*, *miR-34a*, *miR-128*, *miR-145*, *miR-150*, and *miR-335* in oral epithelial dysplasia and normal oral mucosa. We found a consistent increase in mRNA levels of *NOTCH1* in LG and HG dysplasia in comparison with normal mucosa. Similar to *NOTCH1*, mRNA levels of all analyzed CSC markers—*AGR2*, *NANOG*, *OCT4* and *SOX2*—except *KLF4* were also consistently elevated in dysplasia, as were *miR-150* and *miR-128*, with *miR-128* up-regulation observed only in HG dysplasia. We found a positive correlation between expression of *NOTCH1* mRNA with expression of mRNAs of all analyzed CSC markers, as well as with expression of *miR-150*. Immunohistochemical expression of NOTCH1 in oral epithelial dysplasia was variable, showing up-regulation, down-regulation, or expression scores similar to normal mucosa.

To the best of our knowledge, there are no data in the literature on the expression of *NOTCH1* mRNA in oral epithelial dysplasia. Therefore, the potential biological and clinical significance of *NOTCH1* mRNA levels in oral epithelial dysplasia is not known. In contrast to oral dysplasia, increased expression of *NOTCH1* mRNA has previously been demonstrated in a subset of OSCC [17]. That study compared levels of *NOTCH1* mRNA in OSCC and oral lichen planus; normal mucosa was not included. Although oral lichen planus is currently accepted as a potential precancerous lesion, it cannot be directly compared with oral epithelial dysplasia. On the other hand, others have indicated down-regulation of *NOTCH1* mRNA in OSCC [18]. Little is known about the clinical significance of *NOTCH1* mRNA levels in OSCC. Some studies, including our own, have indicated prognostic benefits of higher *NOTCH1* mRNA levels in OSCC [14,19], while others found no association [17].

In contrast to mRNA, expression of NOTCH1 protein in oral epithelial dysplasia, OSCC, and normal oral mucosa has been more extensively studied. In the present study, we found immunohistochemical expression of NOTCH1 in the basal/parabasal cells of normal epithelium, in accordance with other authors [3,4,5,18]. Immunohistochemical expression of NOTCH1 in oral epithelial dysplasia in our study was variable, showing either up-regulation or down-regulation, or expression scores similar to normal mucosa. Previous results on immunohistochemical expression of NOTCH1 in oral epithelial dysplasia in comparison with normal mucosa are also variable. Some authors reported a fairly consistent up-regulation [5] or down-regulation [18] of NOTCH1 in dysplasia, while others reported variable expression, with either a general increase [4] or decrease [3] of NOTCH1.

Clinical significance of NOTCH1 immunohistochemical expression in oral epithelial dysplasia is controversial: various studies have reported increased risk of malignant progression associated with either high [4] or low [3] levels of NOTCH1. In our study, no correlation between expression of *NOTCH1* mRNA and NOTCH1 protein was observed, which might be the result of methodology, but may also reflect complex post-transcriptional regulation of *NOTCH1* expression during oral cancerogenesis. The expression patterns of NOTCH1 in oral epithelial dysplasia found in the present study are in accordance with the complex role of NOTCH in head and neck carcinogenesis, with both oncogenic and tumor-suppressive roles, regulating or being regulated by other factors [2].

It is therefore not surprising that we observed positive correlation between *NOTCH1* mRNA levels and mRNA levels of CSC markers, *ARG2*, *KLF4*, *NANOG*, *OCT4*, and *SOX2*, as well as with *miR-150* in oral epithelial dysplasia. To the best of our knowledge, correlation between *NOTCH1* and CSC markers *ARG*, *KLF4*, *NANOG*, *OCT4*, and *SOX2* in oral epithelial dysplasia has not yet been described. However, we have previously shown that expression of NOTCH1 was correlated with *KLF4*, *NANOG*, and *OCT4* in OSCC [14]. Our observations are supported by other studies. Lee et al. [8] created cells ectopically expressing the Notch intracellular domain and examined self-renewal capacity and CSC marker expression. Constitutive activation of the Notch intracellular domain promoted self-renewal capacity of HNSCC cells and increased expression of CSC markers OCT4, SOX2, and CD44, suggesting that NOTCH1 may be a critical regulator of stemness in HNSCC [8]. Clinical and functional significance of NOTCH1 alterations were analyzed by Pawan et al. in 68 cases of early-stage SCC of the tongue. They showed that *NOTCH1* harbors a low frequency of inactivating mutations and can be amplified and/or overexpressed. HNSCC cell lines overexpressing *NOTCH1*, when plated in the absence of attachment, were enriched in stem cell markers [20].

CSC markers have previously been shown in various studies, including ours, to be important contributors to development of SCC [14,16]. Immunohistochemical analyses of CSC markers have shown elevated expression in oral epithelial dysplasia [21], with limited data about their mRNA expression. In OSCC, we and others have shown that both, *ARG2* and *KLF4* are up-regulated [14,22], and it has been further shown that KLF4 may act both as a tumor suppressor and as an oncogene [22]. Two other studies investigated combined expression of *OCT4* and *SOX2* in oral epithelial dysplasia and OSCC. In the first study, using qPCR, there was a significant difference in *SOX2* and *OCT4* expression between OSCC and oral epithelial dysplasia, with increased expression of *SOX2* and *OCT4* in OSCC, showing correlation with higher tumor grade and with each other [23]. The second study, using immunohistochemistry, investigated association between expression of SOX2, OCT4, and WNT5A in oral epithelial dysplasia, oral SCC, and normal oral mucosa. SOX2 expression was higher in OSCC than in oral epithelial dysplasia and very low in normal oral mucosa. OCT4 was very low in OSCC and oral epithelial dysplasia when compared with SOX2, while negative in normal tissues [24]. Expression of NANOG in oral epithelial dysplasia and OSCC has been more extensively studied. de Vicente et al. immunohistochemically evaluated NANOG expression in oral epithelial dysplasia and OSCC. Their results suggested clinical relevance of NANOG in early stages of OSCC development, and its expression emerged as an early predictor of cancer risk in patients with oral epithelial dysplasia [25]. Similarly, we observed a consistent strong staining for NANOG protein in HG dysplasia and OSCC, with no staining of normal mucosa and very weak staining in LG dysplasia. NANOG protein detection has diagnostic potential for oral HG dysplasia, distinguishing it from LG dysplasia and reactive lesions [16]. In the present study, we also aimed to determine whether NOTCH1 immunohistochemistry could be useful in oral pathology similarly to NANOG, as a diagnostic marker for identifying patients at risk. Even though alterations of NOTCH1 protein in oral epithelial dysplasia and OSCC exist, they are variable and complex, thus making NOTCH1 unreliable as a simple diagnostic marker. Future developments in understanding NOTCH1 regulation may bring new insights into the clinical relevance of NOTCH1 expression patterns in oral epithelial dysplasia and OSCC.

Finally, our study showed up-regulation of *miR-150* and *miR-128* in oral epithelial dysplasia in comparison with normal mucosa, with *miR-128* up-regulation observed only in HG dysplasia. We also observed a strong positive correlation between *NOTCH1* and *miR-150*. No correlation between expression of *miR-150* or *miR-128* and NOTCH1 protein was found. The meaning of these results is currently unclear. Expression of *miR-128* and *miR-150* in invasive OSCC has been previously studied. In some cancers, *miR-150* acts as an oncogene and has been positively correlated with metastasis and tumor recurrence, while in other studies it has been shown to act as a tumor suppressor [26]. For *miR-128*, data are limited, but cell line-based experiments suggest a tumor-suppressive role of *miR-128* in HNSCC [15,27,28]. Similar data based on cell line experiments are found in the literature for *miR-150* [29,30,31,32]. To the best of our knowledge, expression of *miR-150* and *miR-128* in oral epithelial dysplasia has not yet been described in the literature.

## 4. Materials and Methods

### 4.1. Patients and Tissue Samples

Our study included formalin-fixed paraffin-embedded tissue samples from 36 cases of oral epithelial dysplasia and 15 cases of normal oral mucosa. Among the 36 patients with dysplasia, 22 were male and 14 female, aged 43–85 years (64.47 ± 9.78). Samples of normal oral mucosa were obtained from patients surgically treated for non-neoplastic lesions; four patients were males and 11 females, aged 35–86 years (60.67 ± 14.89).

After the surgical procedure, samples were fixed in formalin for 24 h, embedded in paraffin (FFPE), cut at 3–4 µm, stained with hematoxylin and eosin (HE), and analyzed according to standard histopathological procedures. For the purpose of our study, representative samples were selected from the archive of the Institute of Pathology. Representative areas of microscopically normal mucosa and dysplasia on HE slides were chosen and marked for subsequent punching for RNA isolation. We obtained 87 tissue samples in total: 72 samples of dysplasia (2 samples from each patient, one with LG dysplasia and one with HG dysplasia) and 15 samples of normal oral mucosa.

### 4.2. Isolation of RNA

Using a 0.6 mm needle (Manual Tissue Arrayer MTA, Beecher, Estigen, Tartu, Estonia), representative areas were punched from FFPE tissue blocks. After punching, an additional slide was cut, stained with HE, and evaluated by a pathologist. Punching was followed by manual isolation of total RNA using the MagMAX FFPE DNA/RNA Ultra kit (Applied Biosystems; Thermo Fisher Scientific, Foster City, CA, USA). The isolation was performed according to the manufacturer’s instructions with one modification. Briefly, after deparaffinization and xylene removal, protease digestion was performed overnight (300 rpm for 15 s every 4 min). The concentrations of isolated RNAs were assessed fluorometrically on Qubit 3.0 (Applied Biosystems; Thermo Fisher Scientific, Foster City, CA, USA), and purity was analyzed using ND-One.

### 4.3. Reverse Transcription

Total RNA was reverse transcribed (RT) using miScript II RT (Qiagen, Hilden, Germany) according to the manufacturer’s instructions and used to analyze the expression of both mRNAs and miRNAs. The RT reaction volume was 14 µL RT, including 7.94 uL (12 ng/uL) of total RNA, 2.8 µL HiFlex buffer, 1.4 µL 10× Nucleic mix, 1.4 µL of miScript RT enzyme, and 0.46 µL of RNaze inhibitor (Qiagen, Hilden, Germany). The reaction was performed at 37 °C for 60 min and at 95 °C for 5 min (SimpliAmp Thermal Cycler, Applied Biosystems; Thermo Fisher Scientific, Foster City, CA, USA).

### 4.4. Quantitative Real-Time PCR

All quantitative real-time PCR (qPCR) reactions for mRNAs and miRNAs were performed using the ViiA 7 Real-Time PCR System (Applied Biosystems; Thermo Fisher Scientific, Foster City, CA, USA).

Efficiencies of qPCR reactions were calculated using pools of isolated RNAs for each group of samples (normal mucosa, LG dysplasia, HG dysplasia). Reactions were performed in triplicate. For mRNAs, pre-amplified cDNA (see Section 4.4.1) was diluted 5-, 25-, 125-, and 625-fold for each mRNA analysis. For miRNAs, cDNA was diluted 10-, 25-, 100-, 125-, 625-, 1000-, and 3125-fold and used in qPCR reactions, as described below.

#### 4.4.1. Pre-Amplification of mRNAs

Prior to qPCR, pre-amplification of mRNAs was performed using TaqMan PreAmp Master Mix, according to the manufacturer’s instructions (Applied Biosystems; Thermo Fisher Scientific, Foster City, CA, USA). The pre-amplification reaction, with a total volume of 20 µL, included 6.25 µL of cDNA, 6.25 µL of pooled 0.2× TaqMan Gene Expression Assays diluted in Tris-EDTA buffer solution, pH 8.0 (Sigma-Aldrich; Merck KGaA, Darmstadt, Germany), and 12.5 µL 2× TaqMan PreAmp Mastermix (Applied Biosystems; Thermo Fisher Scientific, Foster City, CA, USA). The program of 10 min at 95 °C and 10 cycles of 15 s at 95 °C and 4 min at 60 °C using a SimpliAmp Thermal Cycler (Applied Biosystems; Thermo Fisher Scientific, Foster City, CA, USA).

#### 4.4.2. qPCR for TaqMan Assays

TaqMan assays were used for analyzing the expression of *AGR2*, *KLF4*, *NANOG*, *NOTCH1*, *OCT4* and *SOX2*, which were normalized to *GAPDH*, *IPO8* and *HPRT1* (Table 1). Each qPCR reaction contained 5 µL of 2× TaqMan Gene Expression Master Mix, 4.5 µL of pre-amplified cDNA (fivefold dilution) and 0.5 µL 20× TaqMan Gene Expression Assay (Applied Biosystems; Thermo Fisher Scientific, Foster City, CA, USA). qPCR reactions were performed in duplicate as follows: 50 °C for 2 min, 95 °C for 10 min, and 45 cycles at 95 °C for 15 s and 60 °C for 1 min.

#### 4.4.3. qPCR for miRNAs

For miRNA expression analysis, the miScript SYBR Green PCR Kit (Qiagen, Hilden, Germany) was used. Investigated miRNAs (*miR-27a*, *miR34a*, *miR-128*, *miR-145*, *miR-150*, and *miR-335*) were normalized to *SNORD61* and *SNORD95* (Table 1). qPCR reactions, each with a total volume of 10 µL, contained 5 µL of 2× miScript SYBR Green PCR Mix, 2 µL of cDNA (100-fold dilution), 1 µL 10× miScript Universal Primer, 1 µL 10× miScript Primer, 0.05 µL of ROX dye, and 0.95 µL ddH_2_O. qPCR reactions were performed in duplicate as follows: 95 °C for 15 min and 45 cycles at 94 °C for 15 s, 55 °C for 30 s, and 70 °C for 30 s. At the end of each reaction, melting curves were acquired on the SYBR channel using a ramping rate of 0.7 °C/60 s from 60 to 95 °C.

### 4.5. Immunohistochemical Detection of NOTCH1

For immunohistochemistry, commercially available anti-NOTCH1 antibody (Cell Signaling, cat no. 3608, clone D1E11, dilution 1:200) (Merck, Kenilworth, NJ, USA) was used on unstained 3–4 µm thick slides cut from FFPE tissue blocks. Automated immunostaining was performed using BenchMark ULTRA (Ventana, Tuscon, AZ, USA). For visualization, peroxidase and 3,3′-diaminobenzidine incubation (UltraVIEW DAB Detection Kit, Roche, Basel, Switzerland) was used, followed by counterstaining with hematoxylin.

Reactions were assessed semi-quantitatively. We separately evaluated the extent of staining (less than 5% of cells—score 0, 5–20%—score 1, 21–50% of cells—score 2, 51–80% of cells—score 3, above 80% of cells—score 4) and the intensity of staining (negative—score 0, very weak but higher than background, if present—score 0.5, weak—score 1, moderate—score 1.5, strong—score 2). The final expression score was calculated by multiplying the extent and intensity scores. Cellular localization of staining (cytoplasmic, membranous, nuclear) was also noted.

### 4.6. Statistical Analysis

Data were analyzed according to Latham [33], for both mRNA and miRNA. For each sample, ∆Cq was calculated. The geometric mean of Cqs of reference genes (*IPO8*, *GAPDH*, *HPRT1* for mRNAs; *SNORD61*, *SNORD95* for miRNAs) was first calculated and then subtracted from mRNAs or miRNAs, respectively. The obtained ∆Cq was used for all statistical analyses. For comparison of relative mRNA and miRNA expression between independent groups, the Mann–Whitney U test was used, including correction for multiple comparisons using the Bonferroni method. For correlation analysis between different mRNAs (*NOTCH1* and stem cell markers), as well as between *NOTCH1* and miRNAs, Spearman’s rank correlation coefficient test was used, including calculation of statistical power and estimation of the needed sample size. Statistical analysis of data was performed using IBM SPSS Statistics 27 software (SPSS Inc., Chicago, IL, USA). Differences were considered significant at the cut-off *p* < 0.05 (two-tailed).

## 5. Conclusions

In conclusion, our study showed that, even though *NOTCH1* expression is immunohistochemically variable, its RNA expression suggests that it might contribute to the development of CSC-like properties in oral epithelial dysplasia. Moreover, it might influence the expression of miRNAs, e.g., *miR-150*, which could further regulate a plethora of different genes in oral epithelial dysplasia.

## Figures and Tables

**Table 1:** Primers and probes used in the study.

Name	Catalogue Number/ID	Type of Probe
*GAPDH*	4310884E	TaqMan
*HPRT1*	Hs99999909_m1	TaqMan
*IPO8*	Hs00183533	TaqMan
*AGR2*	Hs00356521_m1	TaqMan
*KLF4*	Hs00358836_m1	TaqMan
*NANOG*	Hs04260366_g1	TaqMan
*NOTCH1*	Hs01062014_m1	TaqMan
*OCT4*	Hs04260367_gH	TaqMan
*SOX2*	Hs04234836_s1	TaqMan
*SNORD61*	MS00033705	SybrGreen
*SNORD95*	MS00033726	SybrGreen
*miR-34a*	MS00003318	SybrGreen
*miR-145*	MS00003528	SybrGreen
*miR-27a*	MS00003241	SybrGreen
*miR-128*	MS00008582	SybrGreen
*miR-150*	MS00003577	SybrGreen
*miR-335*	MS00003976	SybrGreen

## Data Availability

The original contributions presented in this study are included in the article/Supplementary Materials. Further inquiries can be directed to the corresponding author.

## Supplementary Materials

The following supporting information can be downloaded at: https://www.mdpi.com/article/10.3390/ijms27041946/s1.

## Abbreviations

The following abbreviations are used in this manuscript:

CSC	Cancer stem cells
FFPE	Formalin-fixed paraffin-embedded
EMT	Epithelial–mesenchymal transition
HG	High-grade
HNSCC	Head and neck SCC
LG	Low-grade
miRNA	Micro ribonucleic acid
OSCC	Oral SCC
qPCR	Quantitative polymerase chain reaction
SCC	Squamous cell carcinoma

## References

[1] Zhou B., Lin W., Long Y., Yang Y., Zhang H., Wu K., Chu Q. (2022). Notch signaling pathway: Architecture, disease, and therapeutics. Signal Transduct. Target. Ther..

[2] Shi Q., Jiang S., Zeng Y., Yuan X., Zhang Y., Chu Q., Xue C., Li L. (2024). A Notch signaling pathway-related gene signature: Characterizing the immune microenvironment and predicting prognosis in hepatocellular carcinoma. J. Transl. Intern. Med..

[3] Ahmed H., Paterson I., Aziz S.A., Cremona O., Robinson M., Carrozzo M., Valentine R.A. (2023). Expression of Epsin3 and its interaction with Notch signalling in oral epithelial dysplasia and oral squamous cell carcinoma. J. Oral Pathol. Med..

[4] Ding X., Zheng Y., Wang Z., Zhang W., Dong Y., Chen W., Li J., Chu W., Zhang W., Zhong Y. (2018). Expression and oncogenic properties of membranous Notch1 in oral leukoplakia and oral squamous cell carcinoma. Oncol. Rep..

[5] Yoshida R., Nagata M., Nakayama H., Niimori-Kita K., Hassan W., Tanaka T., Shinohara M., Ito T. (2013). The pathological significance of Notch1 in oral squamous cell carcinoma. Lab. Investig..

[6] De Vicente J.C., Lequerica-Fernandez P., Rivas H.T., Blanco-Lorenzo V., Lopez-Fernandez A., Escalante-Narvaez S.A., Herrera I.N.S., Rodrigo J.P., Alvarez-Teijeiro S., Garcia-Pedrero J.M. (2025). Immunohistochemical Evaluation of NOTCH1 Signaling Pathway in Oral Squamous Cell Carcinoma: Clinical and Prognostic Significance. Int. J. Mol. Sci..

[7] Shah P.A., Huang C., Li Q., Kazi S.A., Byers L.A., Wang J., Johnson F.M., Frederick M.J. (2020). NOTCH1 Signaling in Head and Neck Squamous Cell Carcinoma. Cells.

[8] Lee S.H., Do S.I., Lee H.J., Kang H.J., Koo B.S., Lim Y.C. (2016). Notch1 signaling contributes to stemness in head and neck squamous cell carcinoma. Lab. Investig..

[9] Baillie R., Tan S.T., Itinteang T. (2017). Cancer Stem Cells in Oral Cavity Squamous Cell Carcinoma: A Review. Front. Oncol..

[10] Williams H.K. (2000). Molecular pathogenesis of oral squamous carcinoma. Mol. Pathol..

[11] Joseph B.K. (2002). Oral cancer: Prevention and detection. Med. Princ. Pract..

[12] Massano J., Regateiro F.S., Januario G., Ferreira A. (2006). Oral squamous cell carcinoma: Review of prognostic and predictive factors. Oral Surg. Oral Med. Oral Pathol. Oral Radiol. Endodontol..

[13] Dong L., Xue L., Cheng W., Tang J., Ran J., Li Y. (2024). Comprehensive survival analysis of oral squamous cell carcinoma patients undergoing initial radical surgery. BMC Oral Health.

[14] Grubelnik G., Bostjancic E., Groselj A., Zidar N. (2020). Expression of NANOG and Its Regulation in Oral Squamous Cell Carcinoma. BioMed Res. Int..

[15] Gupta P., Chattopadhyay T., Mallick B. (2022). miRNome-transcriptome analysis unveils the key regulatory pathways involved in the tumorigenesis of tongue squamous cell carcinoma. Brief. Funct. Genom..

[16] Grubelnik G., Bostjancic E., Anicin A., Dovsak T., Zidar N. (2020). MicroRNAs and Long Non-Coding RNAs as Regulators of NANOG Expression in the Development of Oral Squamous Cell Carcinoma. Front. Oncol..

[17] Sadeghi E.S., Nematpour F.S., Mohtasham N., Mohajertehran F. (2023). The oncogenic role of NOTCH1 as biomarker in oral squamous cell carcinoma and oral lichen planus. Dent. Res. J..

[18] Sakamoto K., Fujii T., Kawachi H., Miki Y., Omura K., Morita K., Kayamori K., Katsube K., Yamaguchi A. (2012). Reduction of NOTCH1 expression pertains to maturation abnormalities of keratinocytes in squamous neoplasms. Lab. Investig..

[19] Wirth M., Jira D., Ott A., Piontek G., Pickhard A. (2018). High NOTCH1 mRNA Expression Is Associated with Better Survival in HNSCC. Int. J. Mol. Sci..

[20] Upadhyay P., Nair S., Kaur E., Aich J., Dani P., Sethunath V., Gardi N., Chandrani P., Godbole M., Sonawane K. (2016). Notch pathway activation is essential for maintenance of stem-like cells in early tongue cancer. Oncotarget.

[21] Barakat S.M., Siar C.H. (2015). Differential expression of stem cell-like proteins in normal, hyperplastic and dysplastic oral epithelium. J. Appl. Oral Sci..

[22] Li W., Liu M., Su Y., Zhou X., Liu Y., Zhang X. (2015). The Janus-faced roles of Kruppel-like factor 4 in oral squamous cell carcinoma cells. Oncotarget.

[23] Ghazi N., Aali N., Shahrokhi V.R., Mohajertehran F., Saghravanian N. (2020). Relative Expression of SOX2 and OCT4 in Oral Squamous Cell Carcinoma and Oral Epithelial Dysplasia. Rep. Biochem. Mol. Biol..

[24] Vijayakumar G., Narwal A., Kamboj M., Sen R. (2020). Association of SOX2, OCT4 and WNT5A Expression in Oral Epithelial Dysplasia and Oral Squamous Cell Carcinoma: An Immunohistochemical Study. Head Neck Pathol..

[25] De Vicente J.C., Rodriguez-Santamarta T., Rodrigo J.P., Allonca E., Vallina A., Singhania A., Donate-Perez Del Molino P., Garcia-Pedrero J.M. (2019). The Emerging Role of NANOG as an Early Cancer Risk Biomarker in Patients with Oral Potentially Malignant Disorders. J. Clin. Med..

[26] Ameri A., Ahmed H.M., Pecho R.D.C., Arabnozari H., Sarabadani H., Esbati R., Mirabdali S., Yazdani O. (2023). Diverse activity of miR-150 in Tumor development: Shedding light on the potential mechanisms. Cancer Cell Int..

[27] Hauser B., Zhao Y., Pang X., Ling Z., Myers E., Wang P., Califano J., Gu X. (2015). Functions of MiRNA-128 on the regulation of head and neck squamous cell carcinoma growth and apoptosis. PLoS ONE.

[28] Yao Y., Xu Q., Yan L., Jiao Y., Su Q., Li X., Liu C., Zhao F. (2020). MiRNA-128 and MiRNA-142 Regulate Tumorigenesis and EMT in Oral Squamous Cell Carcinoma Through HOXA10. Cancer Manag. Res..

[29] Koshizuka K., Nohata N., Hanazawa T., Kikkawa N., Arai T., Okato A., Fukumoto I., Katada K., Okamoto Y., Seki N. (2017). Deep sequencing-based microRNA expression signatures in head and neck squamous cell carcinoma: Dual strands of pre-miR-150 as antitumor miRNAs. Oncotarget.

[30] Koshizuka K., Hanazawa T., Kikkawa N., Katada K., Okato A., Arai T., Idichi T., Osako Y., Okamoto Y., Seki N. (2018). Antitumor miR-150-5p and miR-150-3p inhibit cancer cell aggressiveness by targeting SPOCK1 in head and neck squamous cell carcinoma. Auris Nasus Larynx.

[31] Wu C., Yang M., Chen H. (2020). Inhibition effect of miR-150 on the progression of oral squamous cell carcinoma by data analysis model based on independent sample T-test. Saudi J. Biol. Sci..

[32] Liu D.K., Yu S., Li J.P., Song W.W., Li J.H. (2021). MiR-150 suppressed cell viability, invasion and EMT via HMGA2 in oral squamous cell carcinoma. Eur. Rev. Med. Pharmacol. Sci..

[33] Latham G.J. (2010). Normalization of microRNA quantitative RT-PCR data in reduced scale experimental designs. Methods Mol. Biol..

